# Research on preventive effect of *Akkermansia muciniphila* AKK PROBIO on acute gouty arthritis in mice

**DOI:** 10.1002/fsn3.4367

**Published:** 2024-08-01

**Authors:** Xin Ma, Na Zhu, Xueping Yu, Wei Wang, Wenzhong Wu

**Affiliations:** ^1^ State Key Laboratory of Bioreactor Engineering East China University of Science and Technology Shanghai China; ^2^ Department of Anesthesia First Affiliated Hospital of Chengdu Medical College Chengdu China; ^3^ Heilongjiang Red Cross Sengong General Hospital Harbin Heilongjiang China

**Keywords:** *Akkermansia muciniphila*, arthritis, expression, inflammation, oxidative stress

## Abstract

In mice with acute gouty arthritis, this study intends to examine the mechanism of action of *Akkermansia muciniphila* AKK PROBIO. We developed a mouse model of acute gouty arthritis using sodium urate. The efficiency and mechanism of AKK PROBIO in preventing acute gouty arthritis in mice were then determined by examining the degree of foot swelling, pain threshold, blood biochemical indicators, histological alterations, and messenger RNA (mRNA) expression changes. The results of the animal experiment showed that AKK PROBIO can lessen mouse foot edema severity and increase pain threshold. AKK PROBIO can enhance the enzyme activity of superoxide dismutase (SOD) and the level of glutathione (GSH) in the ankle joint tissues of mice with acute arthritis while decreasing the enzyme activity of myeloperoxidase (MPO) and the level of malondialdehyde (MDA). Interleukin 6 (IL‐6), interleukin 10 (IL‐10), interleukin 1 beta (IL‐1β), and tumor necrosis factor‐alpha (TNF‐α) levels are all reduced by AKK PROBIO in the blood of mice with acute arthritis. Results from histopathology showed that AKK PROBIO reduced tissue damage in the mouse ankle and foot joints. In the tissues of the ankle joints of mice with acute arthritis, the results of the quantitative polymerase chain reaction (qPCR) and Western blot experiments suggested that AKK PROBIO may inhibit the mRNA and protein expression of extracellular signal‐regulated kinase 1/2 (ERK1/2), cyclooxygenase‐2 (COX‐2), and prostaglandin E_2_ (PGE_2_) in the tissues. AKK PROBIO can also regulate gut microbiota, inhibit harmful bacteria, and enhance valeric acid in the intestine, isobutyric acid, and isovaleric acid. Therefore, it is evident that AKK PROBIO prevents acute gouty arthritis better than glucosamine sulfate. It is a strain that has probiotic potential.

## Graphical abstract

*Akkermansia muciniphila* AKK PROBIO alleviates the symptoms of acute gouty arthritis by increasing the abundance of beneficial gut bacteria such as *Bifidobacteria*, reducing the abundance of *Coprococcus* genus, which is positively associated with gouty arthritis, and regulating the levels of short‐chain fatty acids (SCFAs) in the gut of mice.

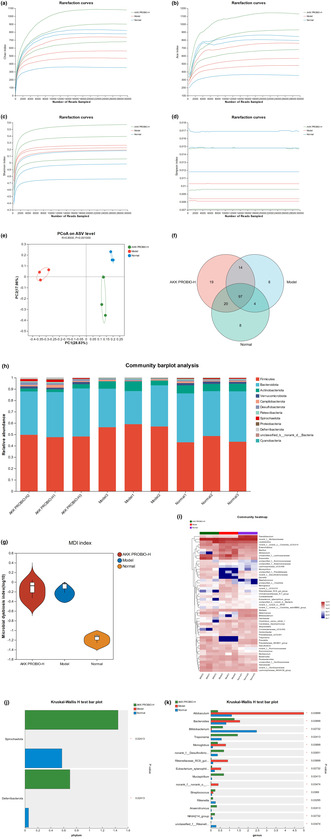

## 1 INTRODUCTION

*Akkermansia muciniphila* is a species of bacteria that naturally resides in the human gut. It is commonly found in the mucus layer of the intestinal lining. The bacteria use mucus as a source of nutrients and have been associated with various health benefits. One of the most well‐known potential health benefits of *Akkermansia muciniphila* is its role in maintaining a healthy gut barrier (Tiwari, 2022). It is thought to contribute to the integrity of the gut lining by promoting the production of specialized proteins that help strengthen the intestinal barrier. A strong gut barrier can prevent harmful substances from leaking into the bloodstream and reduce the risk of inflammation and other gut‐related disorders (Ganesh et al., 2013). *Akkermansia muciniphila* has also been linked to metabolic health. Studies have shown that individuals with a higher abundance of this bacterium in their gut tend to have better metabolic profiles, including lower levels of insulin resistance, improved glucose metabolism, and reduced risk of obesity. Additionally, *Akkermansia muciniphila* has been associated with anti‐inflammatory effects (Tan et al., 2018). It stimulates the production of certain molecules that can help regulate the immune system and reduce chronic low‐grade inflammation, which is believed to play a role in the development of various diseases, including inflammatory bowel disease, type 2 diabetes, and cardiovascular conditions. Although research on *Akkermansia muciniphila* is still relatively new, it has shown promising potential in improving gut and metabolic health (Hansen et al., 2012). However, more studies are needed to fully understand its mechanisms of action and its potential therapeutic applications.

*Akkermansia muciniphila* AKK PROBIO is a newly isolated and confirmed microorganism by our research team. Preliminary testing has shown that it has good in vitro resistance and may have probiotic potential. Therefore, this strain was selected for further animal experiments in this study. In this study, glucosamine sulfate, a medication used to treat and prevent osteoarthritis in various regions of the body, served as a positive control. This study aims to demonstrate the efficacy of AKK PROBIO as a probiotic for the treatment and prevention of arthritis, as well as to confirm the intervention impact it has on gouty arthritis.

## 2 MATERIALS AND METHODS

### 2.1 Experimental strain

A strain of *Akkermansia muciniphila*, named AKK PROBIO, was isolated and identified from the intestines of healthy adults in Suzhou, Jiangsu Province, China. It is the experimental strain for this study. This strain has been preserved at the China General Microbiological Culture Collection Center (CGMCC) and the accession number is CGMCC No. 20955.

### 2.2 Animal experiment

Glucosamine sulfate (positive control, Sigma, CA, USA), normal, model, AKK PROBIO low concentration experiment (AKK PROBIO‐L), and AKK PROBIO high concentration experiment (AKK PROBIO‐H) were the five groups to which 50 male BALB/c mice of specific pathogen‐free (SPF) grade (license number: SCXK (Hu) 2022‐0011, Shanghai Yishang Biotechnology Co., Ltd, Shanghai, China), aged 6 weeks, were randomly assigned. There were 10 mice in each group. The mice in the normal and model groups got 0.1 mL/10 g bw of distilled water orally every day. Glucosamine sulfate was administered orally to the mice in the glucosamine sulfate group at a dosage of 195 mg/kg bw. The AKK PROBIO experimental strains were administered orally to the AKK PROBIO‐L and AKK PROBIO‐H groups for 7 days at dosages of 10^8^ and 10^9^ CFU/kg, respectively. Fifty milligrams of sodium urate salt (2,6,8‐trihydroxypurine, Sigma) was added to 1 mL of phosphate‐buffered saline (PBS) buffer to create a suspension (Zhang et al., 2022). The right tibiotarsal joint (ankle joint) of the remaining mice, with the exception of the model group, was injected with 50 μL of the suspension to cause gouty arthritis. All animals received the relevant samples orally at the same dosages for the following 7 days after waking up from anesthesia. The animal experiment was approved by the Animal Experiment Ethics Committee of Collaborative Innovation Center for Child Nutrition and Health Development with the approval number 2023070045B.

### 2.3 Using hot plate to measure pain threshold

The hot plate method (DB026 intelligent hot plate analyzer, Beijing Zhishu Duobao Biotechnology Co., Ltd., Beijing, China) was used to measure the mice's pain threshold on the last day of the experiment. The mice were placed on a hot plate that was heated to 55°C, which stimulated their paws and caused them to experience discomfort. Each mouse's foot licking activity was painstakingly timed and documented as a sign of how they responded to discomfort. This process was repeated for five different mouse groups, and the length of time that each mouse spent licking its right foot was recorded.

### 2.4 Evaluation of mouse joint swelling

All individuals were given a 6‐h rest time after the hot plate test was used to gauge the mice's pain tolerance. The mice were euthanized using the cervical dislocation technique after that. The diameter of the right ankle was then measured using a digital caliper to determine the degree of ankle swelling.

### 2.5 Oxidative state of mice ankle joint tissues measurement

The 0.1 g of tissue from the right ankle joint of the mouse was weighed in order to determine the tissue's oxidative condition. The tissue was then given 0.9 mL of physiological saline. It was centrifuged at 2012 *g* (revolutions per min) for 10 min to separate the supernatant from the mixture (Long et al., 2022). Using certain test kits, the concentrations of significant indicators, such as myeloperoxidase (MPO), superoxide dismutase (SOD), glutathione (GSH), and malondialdehyde (MDA), in the supernatant of the tissue homogenate were determined (Nanjing Jiancheng Science and Technology Co., Ltd., Nanjing, Jiangsu, China).

### 2.6 Inflammatory cytokine measurement in mouse serum

Retro‐orbital hemorrhage was used to acquire mouse whole blood. To separate the serum, the collected samples were centrifuged at 2012 *g* (revolutions per minute) for 10 min at 4°C. Using enzyme‐linked immunosorbent assay (ELISA) test kits (Thermo Fisher Scientific, MA, USA), the presence of the inflammatory markers IL‐6, IL‐10, IL‐1β, and TNF‐α in the serum was determined.

### 2.7 Mouse tissue histopathological analysis

Mice's ankle joints were removed, and the removed tissue was then preserved in a 10% paraformaldehyde solution. The tissue was then subjected to a 20‐day decalcification process using a solution made of strong hydrochloric acid (HCl), formalin, and distilled water in a 10:9:81 ratio. The tissue samples from the ankle joint were first decalcified, then they were embedded in paraffin, cut into sections, and stained with hematoxylin and eosin (H&E, Beyotime Biotechnology, Shanghai, China). An optical microscope was used to examine the stained slices in order to detect any pathological changes in the tissue (Hu et al., 2022).

### 2.8 mRNA expression analysis in mouse ankle joint tissue

The 0.1 g of tissue was weighed and put in a container to analyze the mRNA expression in ankle joint tissue. After adding it, 0.9 mL of physiological saline was mixed well. The mouse ankle joint tissue was then used to extract RNA using 0.1 mL of RNAzol solution. A microplate spectrophotometer was used to measure the absorbance readings at 260 and 280 nm in order to evaluate the purity and concentration of the RNA, which was then adjusted to 1 μg/L. A reaction system comprising 1 μL of complementary DNA (cDNA) was made after reverse transcription was used to create the cDNA. The reaction mixture comprised 10 μL SYBR Green PCR Master Mix, 7 μL sterile distilled water, and 1 μL each of the forward and reverse primers (Table 1, Thermo Fisher Scientific, USA). Using a quantitative polymerase chain reaction (qPCR) device, the following temperatures were used throughout the reaction: 60 s at 95°C, 15 s at 95°C for 40 cycles, 30 s at 55°C, and 35 s at 72°C. Finally, using glyceraldehyde 3‐phosphate dehydrogenase (GAPDH) as the internal reference, the target gene was examined using the 2‐delta–delta cycle threshold (2^−ΔΔCT^) technique (Zhou et al., 2021).

**TABLE 1:** Primer sequences applied in this study.

Gene	Forward sequence	Reverse sequence
ERK1/2	5′‐TCAAGCCTTCCAACCTC‐3′	5′‐GCAGCCCACAGACCAAA‐3′
COX‐2	5′‐CATCCCCTTCCTGCGAAGTT‐3′	5′‐CATGGGAGTTGGGCAGTCAT‐3′
PGE_2_	5′‐TGGAGGTGAATCCCGTGAGA‐3′	5′‐AAACTCGGTCACCTCCTTGC‐3′
GAPDH	5′‐AGGTCGGTGTGAACGGATTTG‐3′	5′‐GGGGTCGTTGATGGCAACA‐3′

### 2.9 Western blot

After homogenizing 100 mg of each tissue sample with 1 mL of radioimmunoprecipitation assay (RIPA) buffer and 10 mL of phenylmethylsulfonyl fluoride (PMSF), the samples were centrifuged for 5 min at 6037 *g*. The solution for the intermediate protein layer was eliminated. The protein was quantified using a bicinchoninic acid (BCA) protein quantitative kit. Samples were diluted to 50 μg/mL for each group. Sample buffer was then added to the diluted protein at a 4:1 ratio, and the mixture was heated for 5 min at 100°C. The sodium dodecyl sulfate–polyacrylamide gel electrophoresis (SDS–PAGE) separating and concentrating glue was created by proportionately mixing acrylamide, stacking buffer, resolving buffer, distilled water, 10% ammonium persulfate (APS), and TEMED (N,N′,N′‐tetramethylethylenediamine) (Thermo Fisher Scientific). The mixture was then placed onto the runner board for future usage. After the sample and the protein ladder were dotted into the runner plate's sample hole, the protein‐containing SDS–PAGE gel was employed for a 50‐min vertical gel electrophoresis. The transmembrane was sealed with Tris‐buffered saline with Tween 20 (TBST) solution containing 5% skim milk for one hour after the polyvinylidene difluoride (PVDF) membrane (Thermo Fisher Scientific) was activated with methanol for one minute. Following closure, TBST was used to clean the PVDF membrane, and then the primary and secondary antibodies were incubated for two and one hours, respectively, at 25°C. Ultimately, the PVDF film was sprayed with SuperSignal West Pico PLUS and then placed in an iBright FL1000 Imaging System (Thermo Fisher Scientific) for observation.

### 2.10 16S rRNA analysis

The methods for analyzing the microbial composition and diversity of fecal samples involved storing fresh feces in sterile cryotubes, which were then flash‐frozen in liquid nitrogen and transferred to a −80°C freezer for preservation. Total microbial DNA was extracted from mouse fecal samples using the cetyltrimethylammonium bromide (CTAB) method. DNA quantification was carried out using an ultraviolet (UV) spectrophotometer. PCR amplification was performed on the total DNA targeting the V3–V4 regions, with the primer sequences as follows: 338F (5’‐ACTCCTACGGGAGGCAGCA‐3′) and 806R (5’‐GGACTACHVGGGTWTCTAAT‐3′). The amplification program included an initial denaturation (98°C, 30 s), followed by 32 cycles of denaturation (98°C, 10 s), annealing (54°C, 30 s), and extension (72°C, 45 s), with a final extension (72°C, 10 min) and storage at 4°C. The sequencing data analysis was performed using QIIME2.0 (Quantitative Insights into Microbial Ecology).

### 2.11 Determination of short‐chain fatty acids (SCFAs)

After lyophilizing the fecal samples to record the dry weight, saturated sodium chloride (NaCl) solution was added for resuspension. Acidification was performed using a 10% sulfuric acid solution, followed by extraction with anhydrous ether. Anhydrous sodium sulfate was used to remove residual moisture, and the final sample was analyzed using gas chromatography (GC). The analysis employed a gas chromatograph equipped with a flame ionization detector (GC‐FID), with both the injector and FID maintained at 250°C. The flow rates of air, helium, and hydrogen were 400, 30, and 50 mL/min, respectively. The temperature program started at 90°C, held for 1 minute, then rose at a rate of 10°C/min to a final temperature of 190°C, and held for another minute. The split ratio was 40:1, using a polyethylene glycol (PEG) column (30 m × 0.32 mm × 0.25 μm). Concentrations of SCFAs were calculated using an external standard method.

### 2.12 Data evaluation

The trials were carried out in triplicate, and the outcomes were computed by figuring out the average value and standard deviation. The experimental data were presented using the mean and standard deviation. Additionally, a one‐way analysis of variance (ANOVA) was carried out to determine whether there were significant differences (*p* < .05) between the groups.

## 3 RESULTS

### 3.1 Mouse ankle joint swelling intensity

**FIGURE 1:**
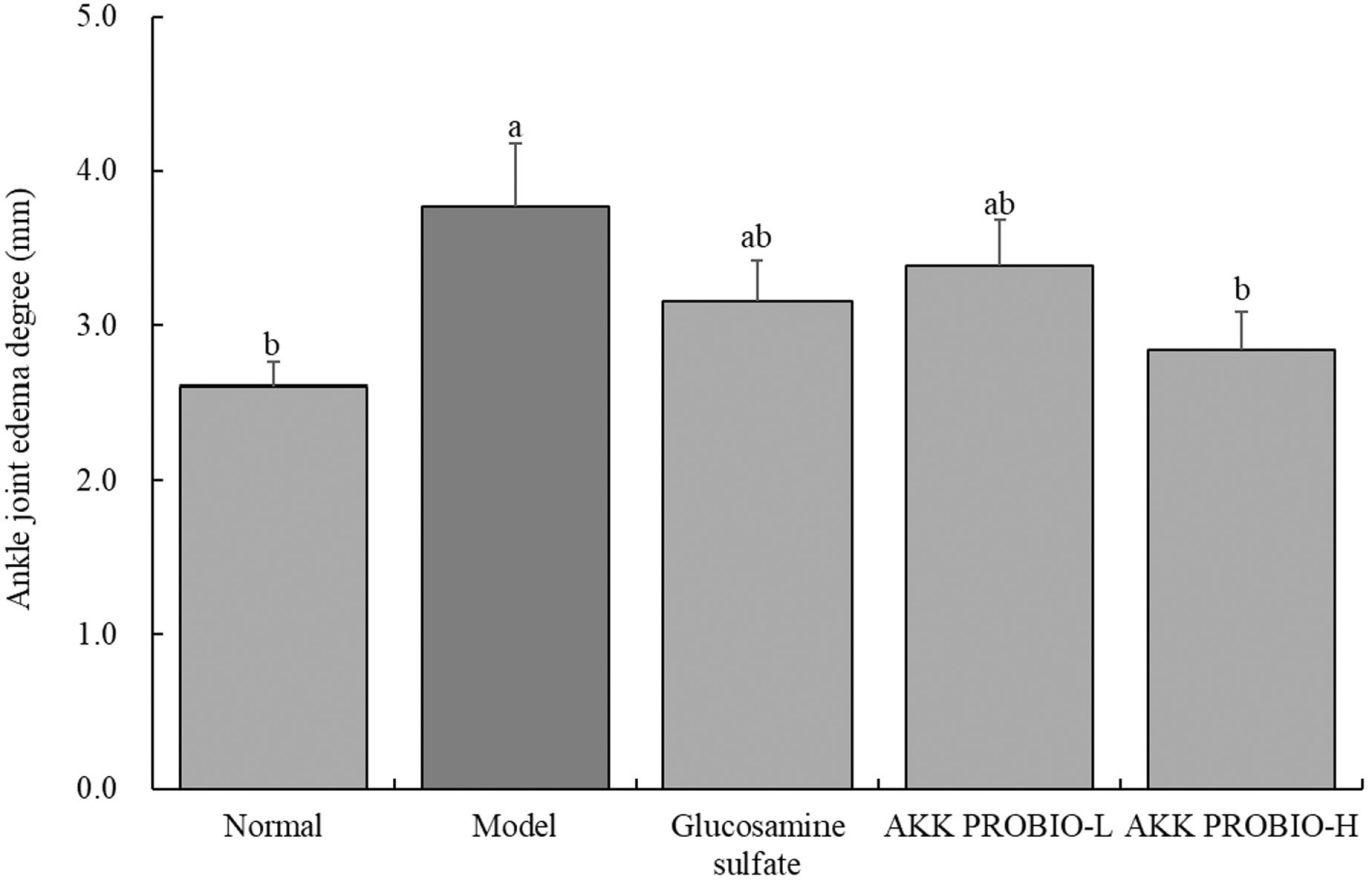
Edema severity in each group of mice's ankle joints. Lowercase alphabet letters that differ from one another indicate a significant difference between the corresponding two groups, whereas lowercase alphabet letters that are the same indicate no significant difference (*p* < .05).

The model group showed the most severe ankle joint edema, as seen in Figure 1. It was shown that the degree of minor ankle joint swelling brought on by arthritis was considerably (*p* < .05) decreased by both glucosamine sulfate and the AKK PROBIO. The strain AKK PROBIO at a high concentration (AKK PROBIO‐H) also demonstrated the best outcomes.

### 3.2 Mouse comfort level

**FIGURE 2:**
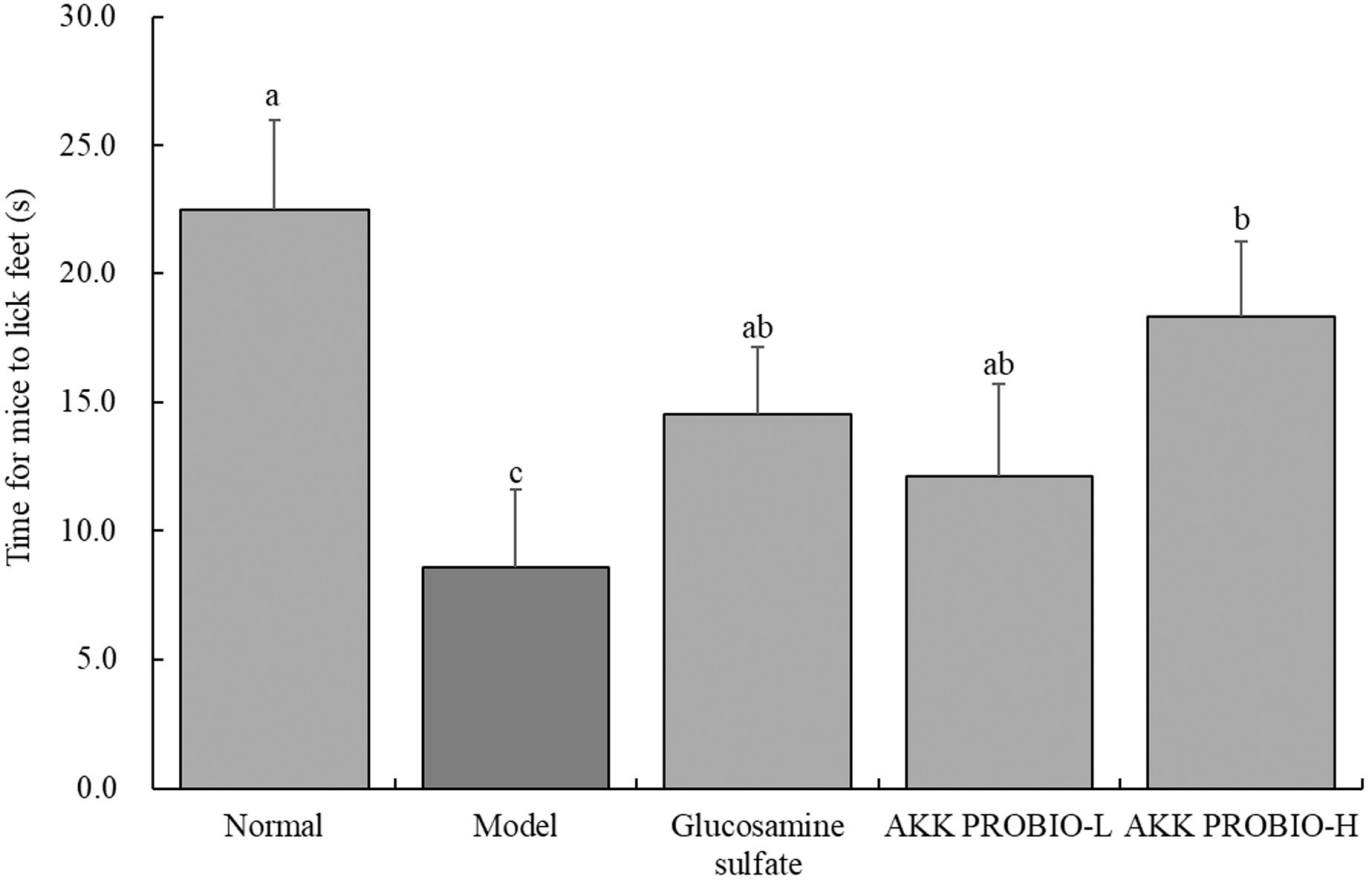
Sensitivity to pain in each group of mice. Lowercase alphabet letters that differ from one another indicate a significant difference between the corresponding two groups, whereas lowercase alphabet letters that are the same indicate no significant difference (*p* < .05).

In contrast to the model group, where it was the shortest, it can be seen from the results shown in Figure 2 that the normal group of mice had the highest pain threshold (time). When compared to the model group, the treatment of glucosamine sulfate and the AKK PROBIO significantly (*p* < .05) raised pain threshold. In addition, glucosamine sulfate and the low concentration of AKK PROBIO (AKK PROBIO‐L) had less of an impact on raising the pain threshold than the high concentration of AKK PROBIO (AKK PROBIO‐H).

### 3.3 Mouse ankle joint tissue MPO activity, SOD enzyme activity, and levels of GSH and MDA

**FIGURE 3:**
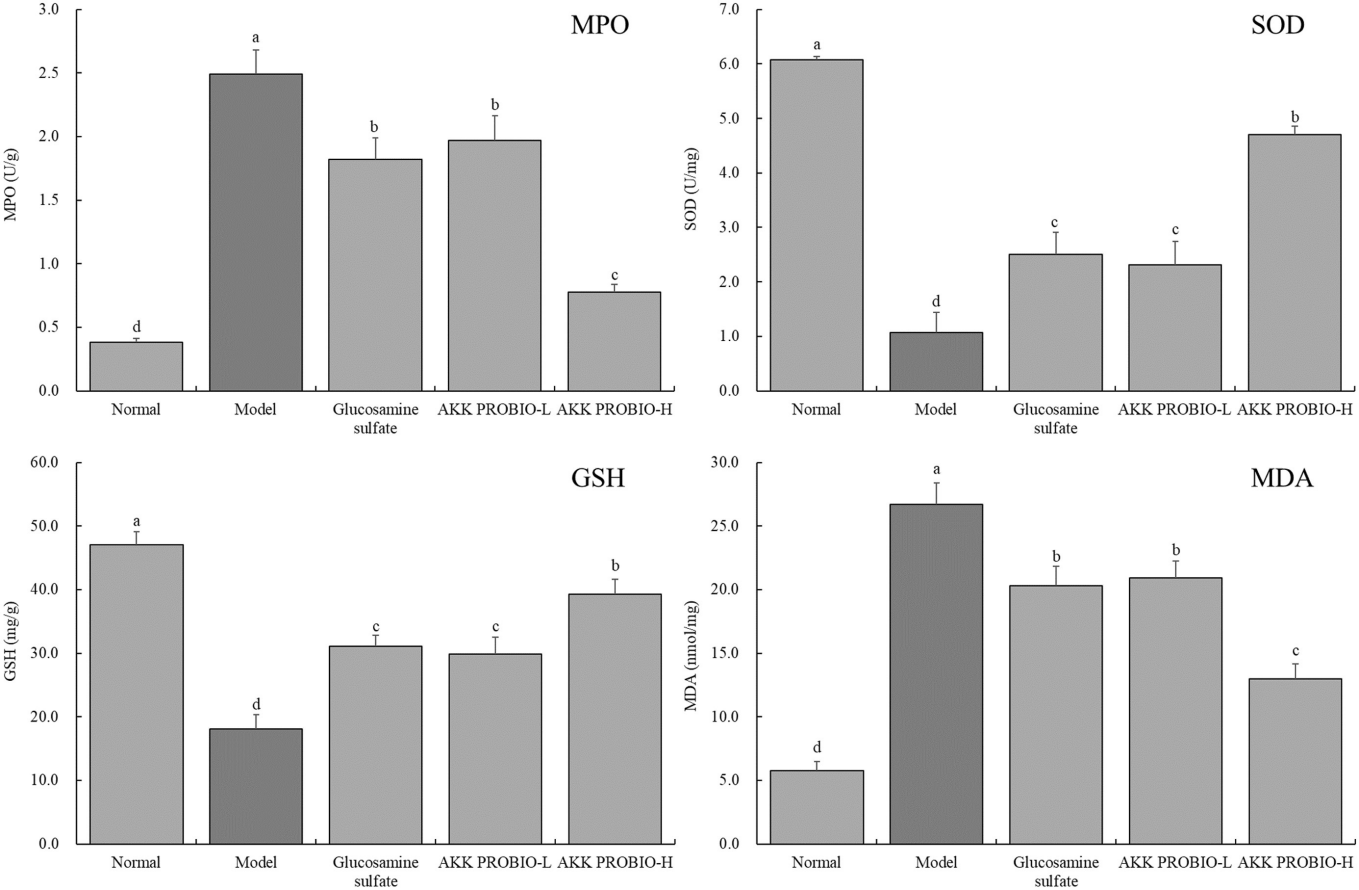
The levels of GSH, MDA, and the MPO, SOD, and SOD enzymes in the mouse ankle joint tissue. Lowercase alphabet letters that differ from one another indicate a significant difference between the corresponding two groups, whereas lowercase alphabet letters that are the same indicate no significant difference (*p* < .05).

The normal group of mice clearly showed the lowest MPO enzyme activity and MDA level in the ankle joint tissue, as well as the greatest SOD enzyme activity and GSH level, according to the findings shown in Figure 3. The ankle joint tissue of the model group of mice, on the other hand, showed the greatest MPO enzyme activity and MDA level while exhibiting the lowest SOD enzyme activity and GSH level. A considerable reduction in MPO enzyme activity and MDA level, as well as an increase in SOD enzyme activity and GSH level, was noticed in the ankle joint tissue of mice with arthritis after oral gavage administration of glucosamine sulfate and the AKK PROBIO. Notably, the strain AKK PROBIO at a high concentration (AKK PROBIO‐H) had the most influence.

### 3.4 Mouse serum IL‐6, IL‐10, IL‐1β, and TNF‐α cytokine concentrations

**FIGURE 4:**
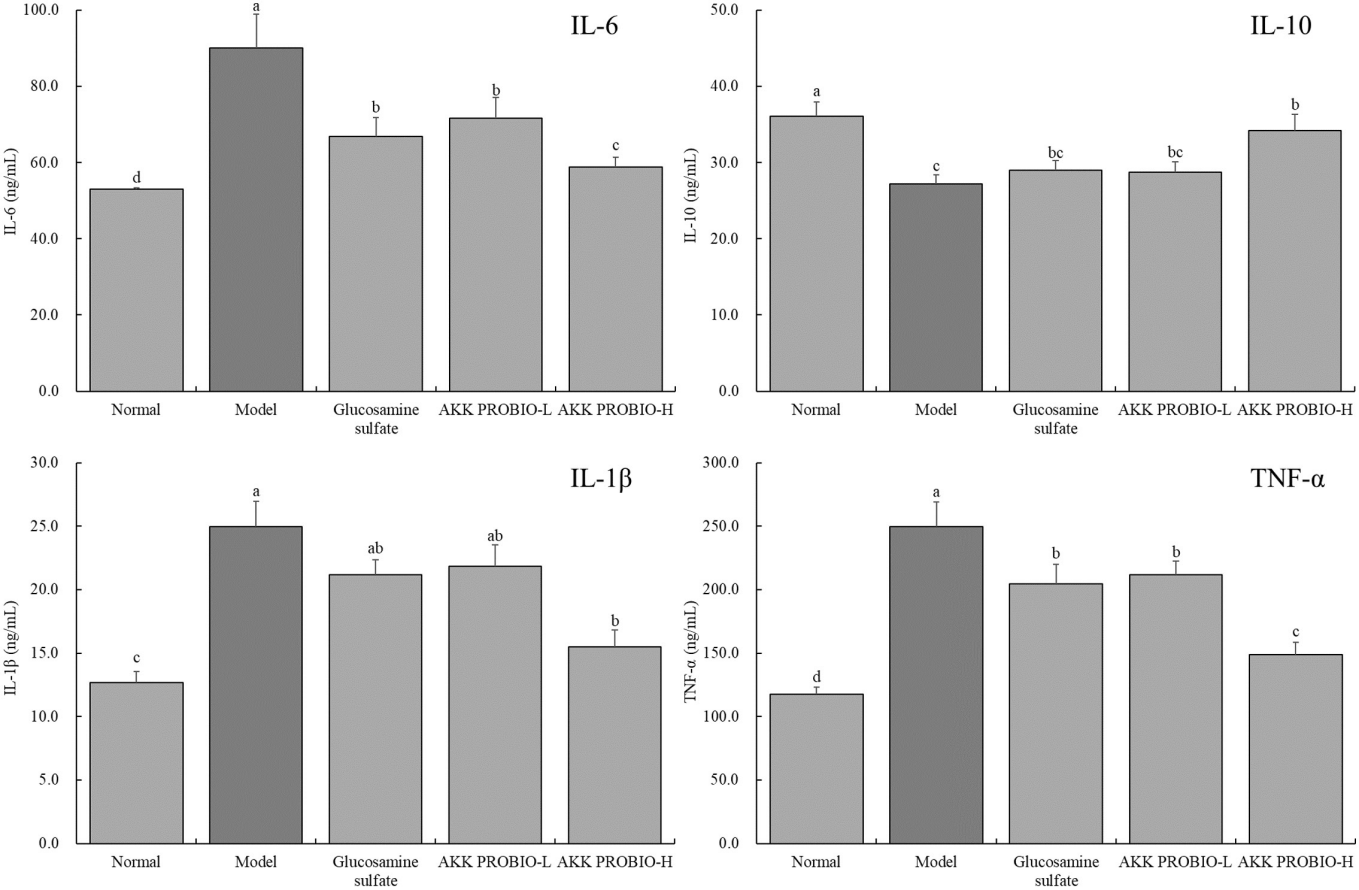
The concentrations of the cytokines IL‐6, IL‐10, IL‐1β, and TNF‐α in mouse serum. Lowercase alphabet letters that differ from one another indicate a significant difference between the corresponding two groups, whereas lowercase alphabet letters that are the same indicate no significant difference (*p* < .05).

Figure 4 makes it clear that the normal group of mice's serum had the lowest concentrations of IL‐6, IL‐1, and TNF‐α. IL‐6, IL‐1β, and TNF‐α cytokine levels, on the other hand, significantly increased in the ankle joint tissue when arthritis was induced in mice (model group) (*p* < .05). IL‐6, IL‐1β, and TNF‐α cytokine levels were significantly decreased by glucosamine sulfate and the AKK PROBIO, with the AKK PROBIO at a high concentration (AKK PROBIO‐H) displaying the best inhibitory effect. Additionally, the model group of mice had the lowest amount of the cytokine IL‐10, whereas the normal group of mice had the greatest level. Both the AKK PROBIO and glucosamine sulfate showed the capacity to raise IL‐10 levels in mouse serum in comparison to the model group, with the AKK PROBIO‐H strain showing the most notable impact.

### 3.5 Histopathological findings in the tissue of the mouse ankle joint

**FIGURE 5:**
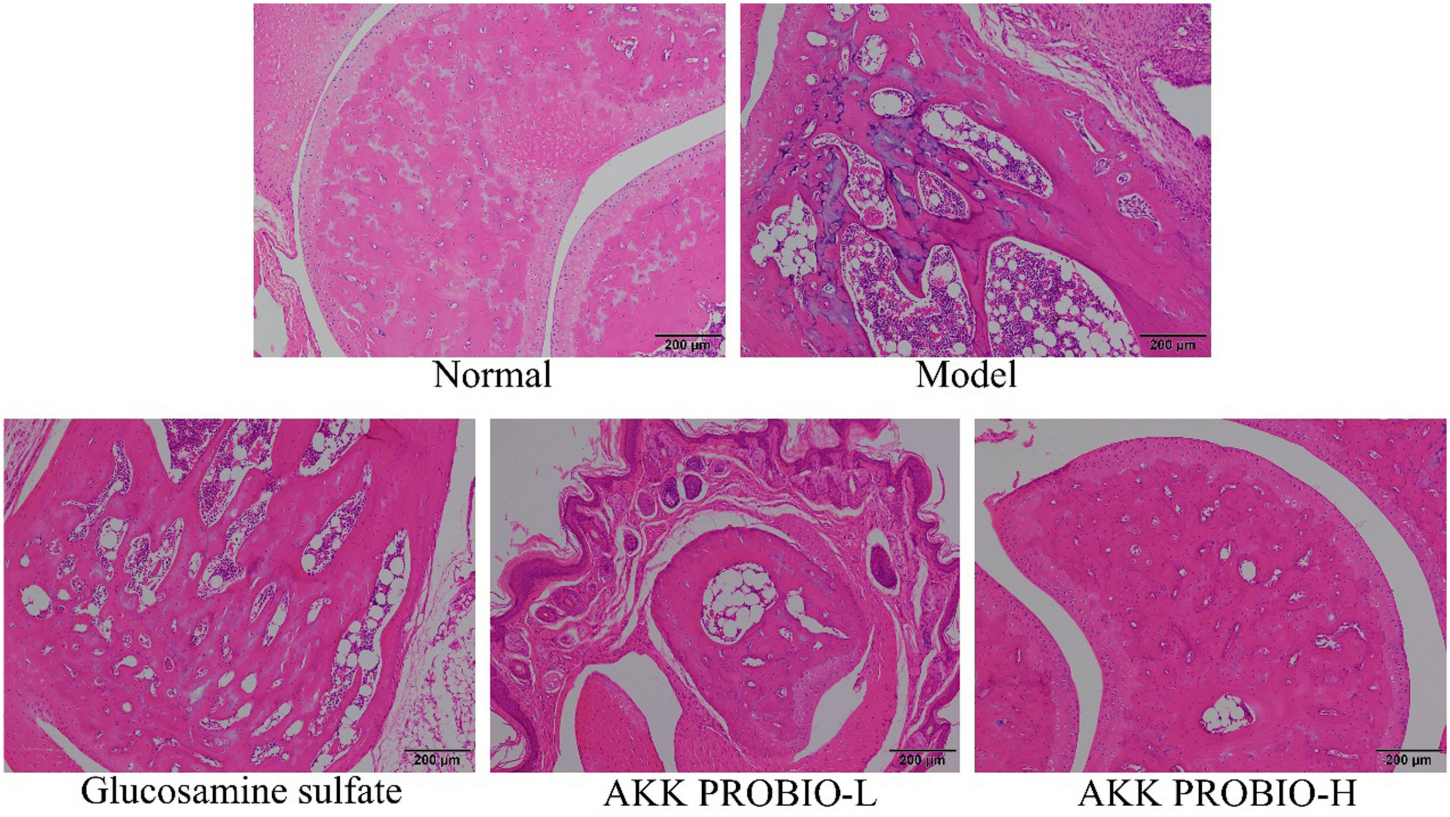
The pathological section observation of mouse ankle joint (×100).

Figure 5's findings make it clear that the model group's ankle joint slices exhibit a marked increase in the infiltration of inflammatory cells. However, glucosamine sulfate treatment and the AKK PROBIO efficiently control the levels of inflammation in arthritic mice, reducing the infiltration of inflammatory cells. Additionally, the inhibitory impact on ankle joint inflammation improves with increasing doses of the AKK PROBIO, with the AKK PROBIO‐H showing greater effectiveness than glucosamine sulfate.

### 3.6 Expression of mRNA and protein in mouse ankle joint tissue

**FIGURE 6:**
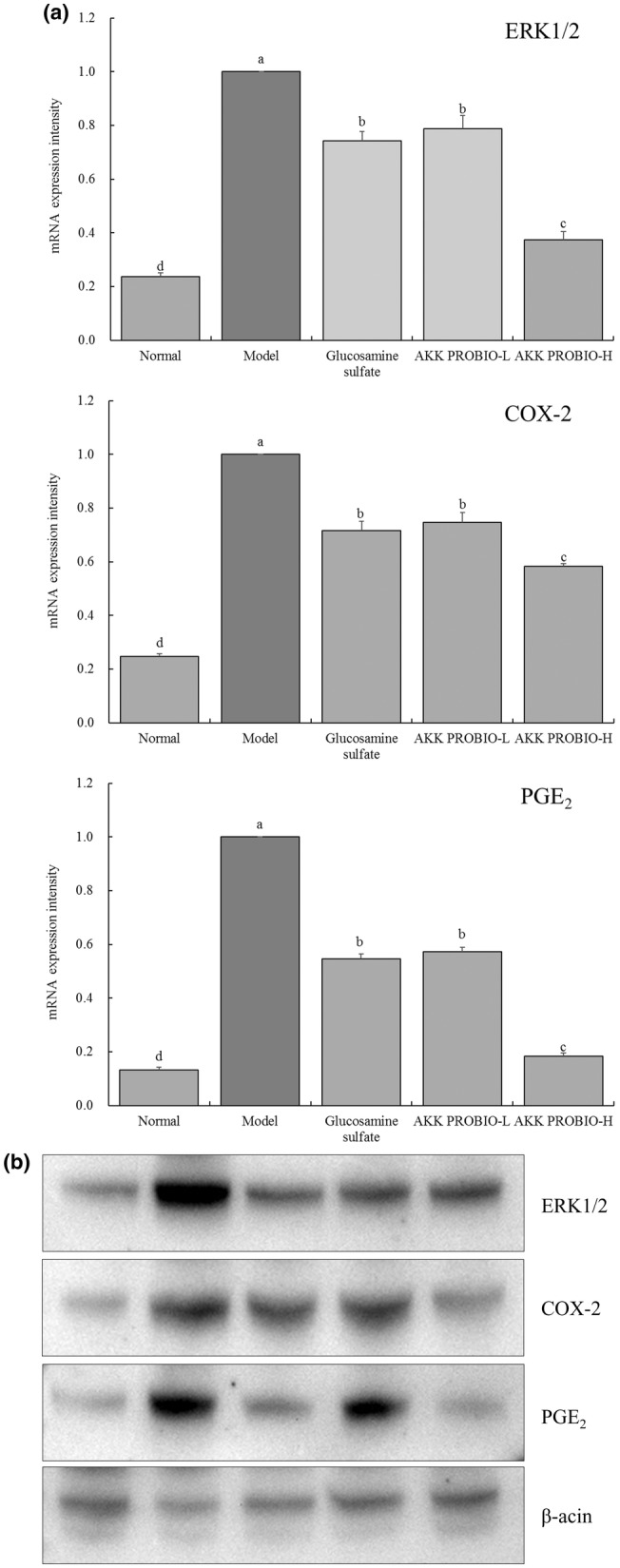
The ERK1/2, COX‐2 and PGE_2_ mRNA (a) and protein (b) expression in mouse ankle joint tissue. Lowercase alphabet letters that differ from one another indicate a significant difference between the corresponding two groups, whereas lowercase alphabet letters that are the same indicate no significant difference (*p* < .05).

Extracellular signal‐regulated kinase 1/2 (ERK1/2), cyclooxygenase‐2 (COX‐2), and prostaglandin E_2_ (PGE2) mRNA expression levels are clearly greatest in the ankle joint tissue of mice in the model group, but they are weakest in the normal group, according to the results shown in Figure 6a. In the joint tissue of arthritic mice, the injection of glucosamine sulfate and the AKK PROBIO significantly (*p* < .05) reduced the mRNA expression of ERK1/2 (0.74‐fold of model), COX‐2 (0.72‐fold of model), and PGE_2_ (0.55‐fold of model). It is noteworthy that the AKK PROBIO has the most capacity to suppress the production of ERK1/2, COX‐2, and PGE_2_, with the AKK PROBIO‐H group showing a more prominent expression (0.37‐, 0.58‐, and 0.18‐fold of model) than the AKK PROBIO‐L group (0.79‐, 0.75‐, and 0.57‐fold of model) and the glucosamine sulfate group. In Figure 6b, the Western blot experiment results showed that the trend of protein expression was consistent with mRNA. The model group showed the highest expression of ERK1/2, COX‐2, and PGE_2_, while the normal group had the weakest expression intensity as mentioned above. The expression of ERK1/2, COX‐2, and PGE2 in the PROBIO‐H group was weaker than those in the AKK PROBIO‐L group and glucose sulfate group.

### 3.7 Changes of intestinal flora in mice α‐diversity index

**FIGURE 7:**
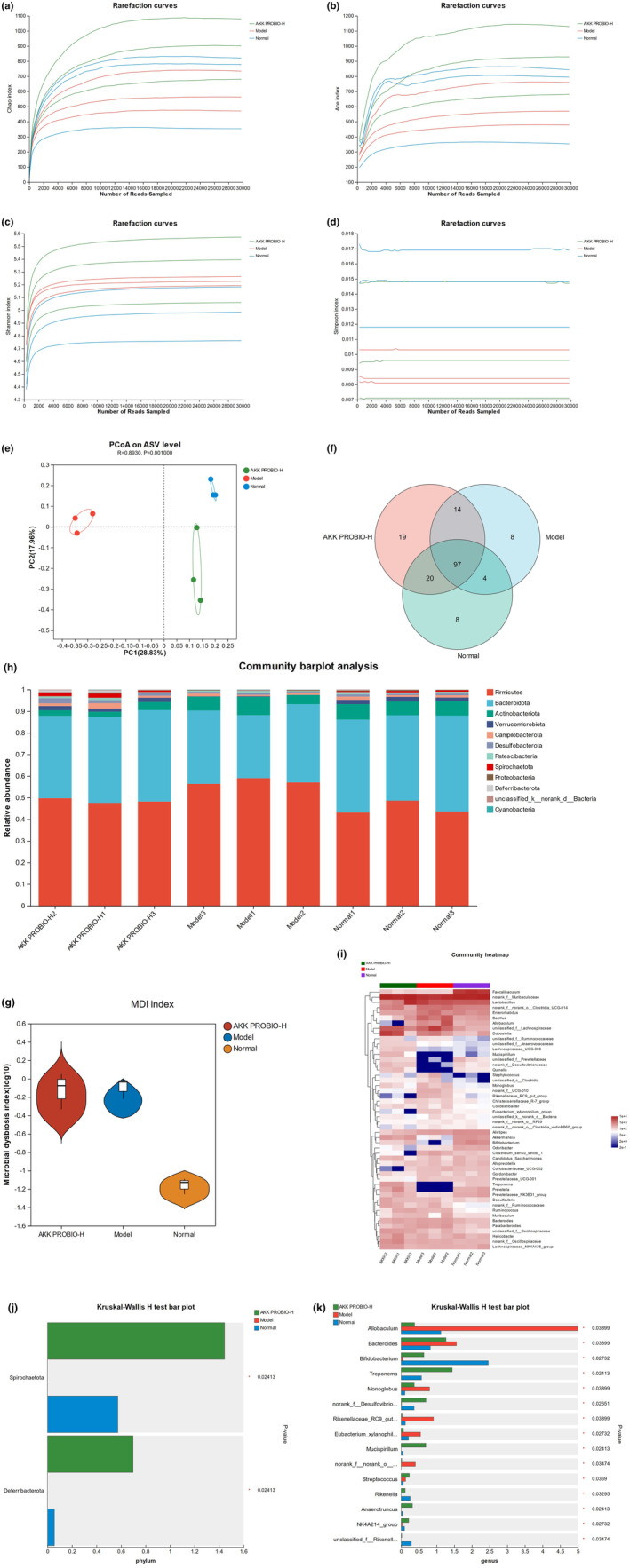
Changes in the gut microbiota of mice in the AKK PROBIO‐H group, model group, and normal group (a). Chao Index (b). Ace Index (c). Shannon Index (d). Simpson Index (e). PCoA based on Unweighted–Unifrac distance matrix (f). Venn diagram at the genus level (g). MDI Index (h). Species composition at the phylum level of the gut microbiota (i). Heatmap of cluster analysis at the genus level of the gut microbiota (j). Top 15 differentially abundant species in the AKK PROBIO‐H group, model group, and normal group.

The results from 16S ribosomal DNA (rDNA) sequencing show that mice treated with AKK PROBIO exhibit increased Chao1, Ace, and Shannon indices and a decreased Simpson Index, indicating an enhancement in the α‐diversity of the gut microbiota in these mice (Figure 3a–d). Principal coordinate analysis (PCoA) reveals that different treatments lead to a complete separation among the gut microbiota of the normal group, model group, and AKK PROBIO‐H group mice, demonstrating significant differences in microbial composition (*p* < .01) (Figure 7e). Compared to the normal and model group mice, the AKK PROBIO‐H group hosts 19 unique microbial taxa and exhibits a lower MDI index relative to the model group (Figure 7f,g). At the phylum level, the abundances of *Firmicutes*, *Bacteroidota*, *Actinobacteriota*, *Verrucomicrobiota*, *Deferribacterota*, *Spirochaetota*, and *Proteobacteria* in the AKK PROBIO‐H group differ from those in the normal and model groups, with significant differences noted only in *Deferibacterota* and *Spirochaetota* (*p* < .05). At the genus level, the abundances of *Allobaculum*, *Bacteroides*, *Monoglobus*, *Rikenellaceae_RC9_gut_group*, and *norank_f_norank_o_Coriobacteriales* in the AKK PROBIO‐H group are significantly (*p* < .05) reduced compared to the Model group, while abundances of *Bifidobacterium*, *Akkermansia*, *Treponema*, *Mucispirillum*, and *unclassified_f_Prevotellaceae* are significantly (*p* < .05) increased (Figure 7h–k).

### 3.8 Determination of the mass concentration of SCFAs in mouse feces

**FIGURE 8:**
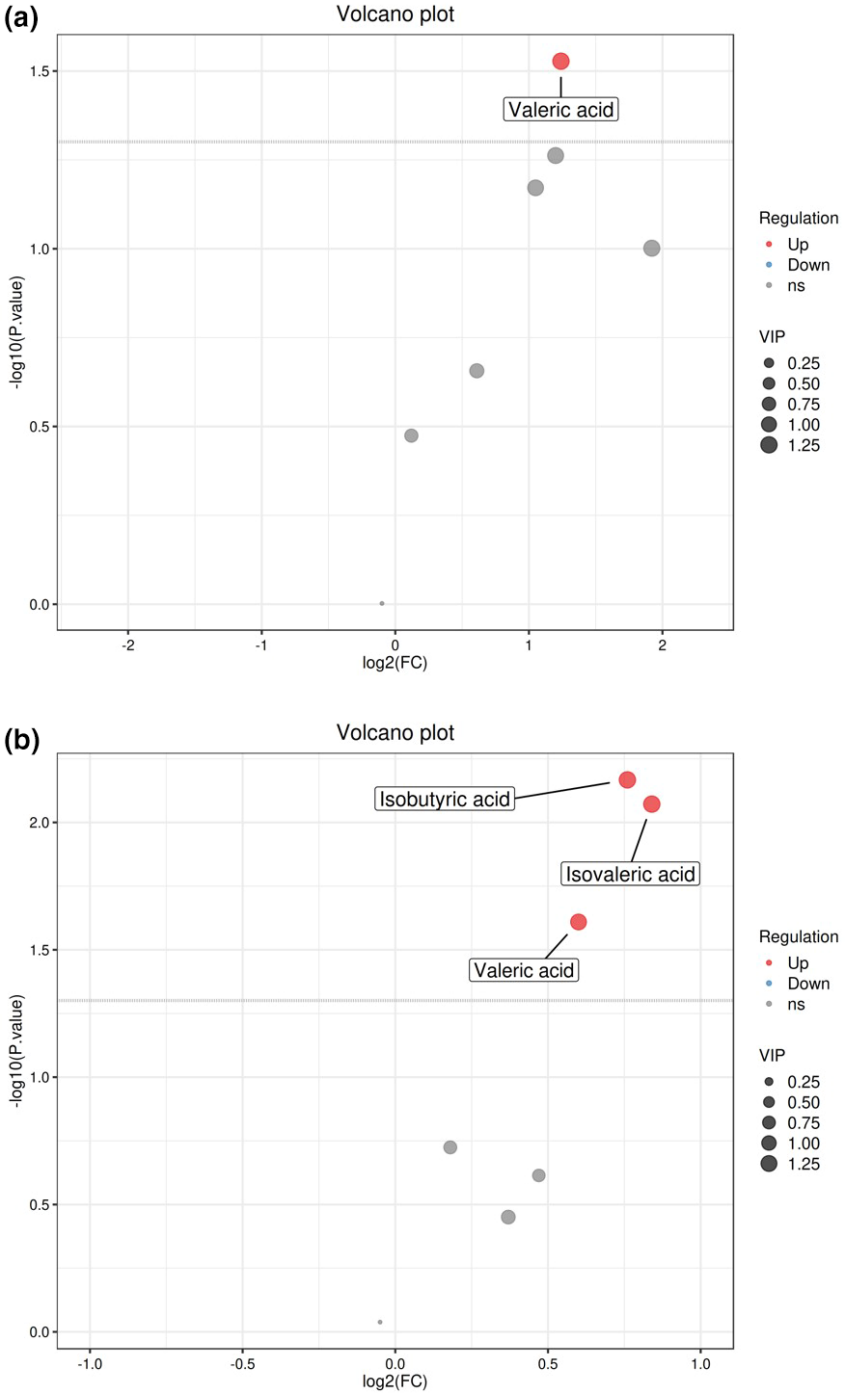
Volcano plots of SCFAs for the AKK PROBIO‐H group compared to the model group (a) and the normal group (b).

As shown in Figure 8, AKK PROBIO affects the SCFAs in the gut of mice with acute gouty arthritis. Compared to the model group, AKK PROBIO increases the content of valeric acid in the gut of mice with acute gouty arthritis. In comparison with the normal group, AKK PROBIO not only enhances the content of valeric acid in the gut of mice but also increases the levels of isobutyric acid and isovaleric acid in the gut of mice with acute gouty arthritis.

## 4 DISCUSSION

The abrupt and intense onset of joint pain that characterizes gouty arthritis causes inflammation in the bursa, cartilage, bone, and other nearby tissues, in addition to the immediate joint capsule. During severe gouty arthritis flare‐ups, the activation of inflammation, the production of different oxygen free radicals, and oxidative stress are all directly related to joint destruction. The increased quantities of oxygen free radicals are mostly attributed to immune and inflammatory processes. In order to effectively manage arthritis, the body's inflammatory response and oxidative stress processes can be controlled (Umar et al., 2012). The host's immune system heavily relies on the gut microbiota and its metabolites. They are closely connected to the formation and progression of arthritis and have a close association with inflammation and unbalanced oxidative stress in the body (Liu & Yu, 2019). Between arthritis sufferers and healthy people, there are noticeable changes in the amount and make‐up of the gut microbiota, according to clinical investigations. A reciprocal interaction between the two components can occur when the dysbiosis of the gut microbiota and the continued advancement of arthritis both affect the incidence and progression of arthritis (Fang & Liu, 2017). According to research, the gut microbiota can affect how well the host's immune system works by controlling immune cell development, using molecular mimicry techniques, and triggering the release of inflammatory mediators, among other processes. The onset of autoimmune disorders may be brought on by these impacts. Probiotics and fecal microbiota transplantation are two methods that have showed promise in reestablishing a balanced gut microbiota, addressing dysbiosis, and perhaps delaying the onset and progression of arthritis as well as easing its accompanying symptoms (Aggarwal et al., 2017).

Characteristic symptoms of arthritis, including joint pain, swelling, and limited movement, are seen during the acute phase of the disease. Several studies have used techniques, such as examining foot swelling and assessing pain threshold in experimental animals, to assess the severity of arthritis (Teranaka et al., 2014). Therefore, in this investigation, the pain threshold was assessed using a hot plate analgesia meter and the measurement of mouse foot edema was carried out by ocular observation. The experiment's outcomes have further shown that AKK PROBIO successfully avoids foot swelling in mice and lessens the decrease in pain threshold brought on by acute arthritis.

Reactive oxygen species (ROS) and oxidative stress responses can be sparked by the oxidative characteristics of myeloperoxidase (MPO). ROS can exacerbate inflammation and injury in joint tissues by causing oxidative damage to cells and tissues. MPO can also stimulate the synthesis of inflammatory mediators including cytokines and chemotactic factors, which will amplify the inflammatory response in arthritic conditions. In arthritis, oxidative stress brought on by inflammation causes an excessive amount of intracellular ROS to be produced, outpacing the antioxidant capacity of the cell. These ROS can harm joint tissues and aggravate arthritis‐related pain and inflammation (Barlow & Bucknall, 1988).

In order to scavenge ROS, lessen oxidative stress, and shield joint tissues from ROS‐induced damage, superoxide dismutase (SOD) and glutathione (GSH) are essential (Wetscher et al., 1995). In arthritis, oxidative stress and inflammation can both enhance lipid peroxidation, which increases the generation of malondialdehyde (MDA). MDA can harm joint structures and worsen arthritis symptoms and inflammation if it builds up too much in the body. Additionally, according to a study (Yuan et al., 2014), MDA can stimulate the production of inflammatory cells and mediators, which will accelerate the breakdown of bone, cartilage, and joint tissues. The findings of the experiment show that AKK PROBIO's capacity to reduce oxidative stress may be a key strategy for avoiding acute arthritis.

Body tissue damage is significantly influenced by inflammatory cytokines. As a result of tissue oxidation and inflammation brought on by pathological alterations, numerous inflammatory mediators are released during oxidative stress. Tissue damage can be made worse by abnormal elevations in pro‐inflammatory cytokines, such as IL‐6, IL‐10, IL‐1β, and TNF‐α (Tripathy et al., 2021). A cytokine known as IL‐6 is essential to the inflammatory process. It increases the production of inflammatory mediators including IL‐1β and TNF‐α, which exacerbates joint inflammation, in synovial cells and other inflammatory cells. Additionally, IL‐6 encourages angiogenesis and synovial cell growth, which exacerbates inflammation and tissue loss. T cells, B cells, and macrophages are all impacted by the regulation of IL‐6 on immune cell development and operation. According to a research (Gottenberg et al., 2012), excessive IL‐6 synthesis in rheumatoid arthritis can activate and multiply inflammatory T cells and B cells, elicit immunological responses, and lead to the formation of autoantibodies, aggravating the condition. An anti‐inflammatory cytokine called IL‐10 is principally in charge of preventing inflammation and preserving the equilibrium of the immune system. Inflammatory mediators like IL‐1β and TNF‐α are suppressed by IL‐10, which lessens the severity of inflammatory responses and reduces arthritic symptoms and inflammation. On the other hand, a lack of IL‐10 might result in increased immune cell activation and long‐lasting inflammatory reactions, aggravating the course of arthritis (Quattrocchi et al., 2001). Since it triggers an inflammatory response and encourages the activation and proliferation of inflammatory cells in joints, IL‐1β plays a crucial role in the onset of arthritis. Joint inflammation is exacerbated by IL‐1β because it causes synovial and cartilage cells to produce additional inflammatory cytokines including TNF‐α and IL‐6. The swelling, discomfort, and functional impairment that come from increased joint inflammation are caused in part by these inflammatory agents. Further joint degeneration is brought on by IL‐1β's promotion of osteoporosis and cartilage destruction in joints (Czerny et al., 2010). By bringing inflammatory cells to the joints and encouraging their activation and proliferation, TNF‐α causes inflammation. It intensifies the inflammatory response in the joints and causes joint swelling, discomfort, and functional impairment by inducing synovial and cartilage cells to generate more inflammatory mediators, such as IL‐1β, IL‐6, and interleukin‐8 (IL‐8) (Redlich et al., 2002). The experimental findings of this study also show that AKK PROBIO has the capacity to control inflammatory cytokines, acting as an acute arthritis preventative agent.

The COX‐2/PGE2 pathway and the ERK1/2 signaling pathway collaborate. Upon activation, ERK1/2 can control COX‐2 expression and activity either directly or indirectly, which has an effect on PGE2 production. A cell signaling molecule with a long history of research, ERK1/2 plays a role in a number of biological functions, including cell division, proliferation, and inflammation. In arthritis, ERK1/2 activation levels are frequently markedly elevated, which may be related to the growth of inflammatory cells, the production of inflammatory mediators, and damage to the tissues of the joints (Marotte et al., 2010). An essential enzyme in the production of prostaglandins, particularly PGE2, during the inflammatory response is COX‐2. In arthritis, COX‐2 expression is frequently upregulated, which increases PGE2 generation (Kumar et al., 2015). According to the experiment (Bouffi et al., 2010), PGE2 has a pro‐inflammatory function in arthritis and contributes to pathological processes, such as pain associated with the condition, the activation of inflammatory cells, and skeletal degradation. Effectively controlling joint tissue mRNA, AKK PROBIO modifies the expression of genes associated to arthritis in a way that promotes the body's ability to heal. This emphasizes its significance in the regulation of acute arthritis.

From the analysis of the 16S rDNA data, we can observe that treatment with sodium urate salt significantly reduces the abundance of the gut microbiota in mice. However, the intervention with AKK PROBIO can alleviate, and even neutralize, the adverse effects caused by sodium urate salt on the gut microbiota. The analysis was performed using PCoA based on the Unweighted–Unifrac distance, selecting the principal coordinate combinations with the highest contribution rates for graphical demonstration. If the sample distances are closer, it signifies more similar species composition structures. The treatments with sodium urate salt and AKK PROBIO cause significant separation of the microbial structure in the gut of the AKK group and the model group from the normal group. *Bifidobacteria* are crucial components of beneficial intestinal bacteria and, in cooperation with gut microbiota, provide the body with nutrients, inhibit the growth of harmful bacteria, maintain the intestinal biological barrier, promote intestinal immune balance, and maintain human health (Reyed, 2007). Studies have shown that oral administration of *Bifidobacterium breve*, *Bifidobacterium longum*, and *Bifidobacterium bifidum* can prevent the occurrence of arthritis in Adjuvant‐Induced Arthritis (AIA) rats (Achi et al., 2019). *Akkermansia muciniphila*, a strictly anaerobic gut bacterium isolated from feces, is the first gut bacterium successfully isolated and identified from the phylum *Verrucomicrobia*. It can improve the inflammatory response and alleviate insulin resistance and glucose intolerance in obese and diabetic patients. An appropriate abundance of *A. muciniphila* colonization can also promote the development of the host's immune system, thereby promoting intestinal health and maintaining metabolic balance (Mithieux, 2018). A study indicated that in the intestines of patients with gout, there is a significant increase in the genus *Bacteroides* and its related taxa including the class *Bacteroidia*, the order *Bacteroidales*, and the family *Bacteroidaceae* (Xing et al., 2015). In this experiment, the abundance of *Bacteroides* in the model group was also increased, whereas AKK PROBIO was able to mitigate this trend.

Short‐chain fatty acids (SCFAs), defined as carboxylic acids with fewer than six carbon atoms, are vital metabolic byproducts produced by gut microbiota. These acids serve as key energy sources for colon and ileum cells, and they play a crucial role in regulating the barrier and defense functions of intestinal epithelial cells. The three predominant SCFAs in the gastrointestinal tract are acetate, propionate, and butyrate. As the endpoint products of polysaccharide fermentation by bacteria, these acids are essential components of the gut environment, contributing to its regulatory functions and maintaining the physiological state of the colon. SCFAs facilitate this by lowering the gut pH, which inhibits the replication of pathogenic bacteria and suppresses the onset of infectious diseases (Morrison & Preston, 2016). Moreover, SCFAs modulate innate immunity by influencing the recruitment of immune cells, such as macrophages, neutrophils, and dendritic cells, to peripheral areas (Tan et al., 2014). The role of valeric acid in intestinal health remains less understood; however, preliminary studies suggest that it may stimulate the growth of intestinal epithelial cells and could potentially benefit conditions, such as colitis, cardiac metabolic disorders, and cancer (Onrust et al., 2018; Yuille et al., 2018). Isovaleric and isobutyric acids, produced from the fermentation of branched‐chain amino acids (e.g., valine, leucine, and isoleucine) or aromatic amino acids (e.g., tyrosine and phenylalanine), also play significant yet complex roles in both intestinal and metabolic health. Although these amino acids are generally considered detrimental, their presence is closely linked with the gut microbiota's composition and function (Bourdeau‐Julien et al., 2023). We hypothesize that AKK PROBIO alleviates the symptoms of acute gouty arthritis by increasing the abundance of beneficial gut bacteria, such as *Bifidobacteria*, reducing the abundance of *Coprococcus* genus, which is positively associated with gouty arthritis, and regulating the levels of short‐chain fatty acids in the gut of mice.

In this work, AKK PROBIO was discovered to be an efficient pain reliever and ankle swelling reducer in mice. AKK PROBIO might reduce oxidative stress and inflammation in the ankle joints. AKK PROBIO has been found to effectively regulate gut microbiota, increase beneficial bacteria, and inhibit harmful bacteria, while increasing the content of SCFAs in the intestine, thereby exerting an intervention effect on acute gouty arthritis. The experimental results provide important insights into the production of high‐quality probiotics for intervention in arthritis. Additional clinical research is still needed to confirm these findings, and additional investigation of AKK PROBIO is called for in the future.

## AUTHOR CONTRIBUTIONS

**Xin Ma:** Writing – original draft (equal). **Na Zhu:** Writing – original draft (equal). **Xueping Yu:** Data curation (equal). **Wei Wang:** Writing – review and editing (equal). **Wenzhong Wu:** Writing – review and editing (equal).

## ACKNOWLEDGEMENTS

None.

## FUNDING INFORMATION

This work was supported by Suzhou Social Development Science and Technology Innovation Project (2022SS35).

## CONFLICT OF INTEREST STATEMENT

The authors declare that they have no conflicts of interest.

## Data Availability

All data generated or analyzed during this study are included in this article. The datasets used and/or analyzed during the current study are available from the corresponding author on reasonable request.
